# Role of antioxidant enzymes and small molecular weight antioxidants in the pathogenesis of age-related macular degeneration (AMD)

**DOI:** 10.1007/s10522-013-9463-2

**Published:** 2013-09-22

**Authors:** Paulina Tokarz, Kai Kaarniranta, Janusz Blasiak

**Affiliations:** 1 Department of Molecular Genetics, Faculty of Biology and Environmental Protection, University of Lodz, Pomorska 141/143, 90-236 Lodz, Poland; 2 Department of Ophthalmology, Institute of Clinical Medicine, University of Eastern Finland, Kuopio, Finland; 3 Department of Ophthalmology, Kuopio University Hospital, Kuopio, Finland

**Keywords:** AMD, Oxidative stress, Antioxidant enzymes, Small molecular weight antioxidants, ROS, Retinal pigment epithelium

## Abstract

Cells in aerobic condition are constantly exposed to reactive oxygen species (ROS), which may induce damage to biomolecules, including proteins, nucleic acids and lipids. In normal circumstances, the amount of ROS is counterbalanced by cellular antioxidant defence, with its main components—antioxidant enzymes, DNA repair and small molecular weight antioxidants. An imbalance between the production and neutralization of ROS by antioxidant defence is associated with oxidative stress, which plays an important role in the pathogenesis of many age-related and degenerative diseases, including age-related macular degeneration (AMD), affecting the macula—the central part of the retina. The retina is especially prone to oxidative stress due to high oxygen pressure and exposure to UV and blue light promoting ROS generation. Because oxidative stress has an established role in AMD pathogenesis, proper functioning of antioxidant defence may be crucial for the occurrence and progression of this disease. Antioxidant enzymes play a major role in ROS scavenging and changes of their expression or/and activity are reported to be associated with AMD. Therefore, the enzymes in the retina along with their genes may constitute a perspective target in AMD prevention and therapy.

## Introduction

Age-related macular degeneration (AMD) is a progressive disease of the central part of the retina, which may lead to a partial or complete vision loss in one or both eyes among people aged 55 years and older in developed countries. The disease may be accompanied by the reduction of the visual acuity, however, the absence of visual impairment does not exclude AMD (Bird et al. 1995). The major pathological changes associated with AMD are observed in the functionally and anatomically related tissues, including photoreceptors, retinal pigment epithelium (RPE), Bruch’s membrane and choriocapillaries (Bhutto and Lutty 2012). Classically, two subgroups of AMD are distinguished, atrophic (dry, non-exudative) AMD, characterized by the degradation of RPE and secondary photoreceptors in the macular area and as a consequence the accumulation of extracellular deposits denoted drusen between the RPE and Bruch’s membrane; and exudative (wet, neovascular) AMD associated with choroidal neovascularisation (CNV), which may cause the detachment of RPE or retina, exudation, haemorrhages, inflammation and scar tissue formation in the retina (Bird et al. 1995). The most common form of advanced AMD is the dry one, but it may progress to the wet form, which contributes to rapid loss of vision (Fine et al. 2000). The wet form of AMD occurs less frequently (15 %) than the dry one (85 %), but it accounts for two-third of individuals who have significant visual loss, according to macular degeneration association estimates (MDA 2013). AMD is the third cause of blindness globally and the primary cause (approximately 50 % of legal blindness incidence) in industrialized countries as reported by World Health Organization (WHO) (2013). According to the National Eye Institute (NEI) calculations, there is a higher prevalence of AMD in white people than in other races, and that this disease is more common in women (65 vs. 35 % in men in US in 2010) (NEI 2013). In addition, the number of AMD incidence has increased by 18 % since 2000 till 2010 and is expected to double by the year 2020. Since AMD is uncommon among people under the age of 50, the increase in the absolute number of affected people globally may be a consequence of population aging. Initiation and progression of AMD may be induced by genetic, epigenetic and environmental risk factors. Apart from positive correlation of the disease with age, other risk factors are prevalent, the most important being cigarette smoking, white race, female sex, blue iris colour, obesity, nutritional factors and insufficient antioxidants in the diet (Kaarniranta et al. 2011). However, the pathogenesis of AMD is still elusive, likely due to its multifactorial etiology. It is believed that the senescence of RPE cells and Bruch’s membrane, the impaired blood flow in the vascular membrane of eye, the retina exposure to UV and blue light and the genetic predisposition play a significant role in the development of AMD (Majji et al. 2000; Tanito et al. 2007). Also, oxidative stress is believed to contribute to the pathogenesis of AMD and its role in generating cellular damage in RPE cells and choriocapillaris is well documented (Lu et al. 2006). It is presumed that the loss of RPE cells is an early event in AMD (Dorey et al. 1989). The RPE cells degradation is mainly attributed to oxidative stress, which may be a consequence of attenuated antioxidant cell defense systems or augmented level of ROS (Justilien et al. 2007). Oxidative stress, generated by the oxygen-rich environment and the exposure to light in the eye, modifies the compounds in the photoreceptors, which are then shed in the form of photoreceptor outer segments (POS) and phagocyted by RPE cells (Beatty et al. 2000). RPE consists of postmitotic cells, which are thus deprived of the ability to propagate (Klein et al. 1990). Due to this, RPE cells accumulate damage during the life-span and the extent of such changes increases with age (Cai et al. 2000). These changes include the dysfunction of RPE cells metabolism and insufficiency in their phagocytic function (Chen et al. 2009a). The depletion of these protective mechanisms in RPE cells may lead to the accumulation of toxic photoproducts and further generation of ROS. The increasing concentration of ROS may lead to damage to organelles, including mitochondria and lysosomes (Chen et al. 2009a; Blasiak et al. 2013). The process of ROS formation at the mitochondria is known as the vicious cycle, in which one process stimulates the other (Blasiak et al. 2013). Besides lysosomal degradation, other cell clearance systems, including autophagy, may be altered in AMD (Kaarniranta et al. 2013). The resulting decrease in cellular components degradation propels lipofuscinogenesis (Krohne et al. 2010). Elevated level of undigested or insoluble material in the form of lipofuscin may induce apoptosis (Sparrow et al. 2000). Since RPE cells are postmitotic, their death results in the reduction of RPE cell density in the RPE layer (Del Priore et al. 2002). Thus the remaining RPE cells face a higher number of ROS. This increases oxidative stress in RPE cells promoting pathogenic processes (Strauss 2005). All these processes are enhanced by the age-dependent decline in the level of antioxidants, the most significant being α-tocopherol (Friedrichson et al. 1995). Also hypopigmentation—a noticeable sign of melanosomes photobleaching—augments in the age-dependent manner (Feeney-Burns et al. 1984). The reduction in the number of melanosomes combined with the attenuation of their photo-protective function may propel the progression of AMD (Zadlo et al. 2007). When RPE cells become insufficient to store shed POS or when RPE cells are degraded, POS may be stored between Bruch’s membrane and RPE layer as drusen (Strauss 2005). The negative effect of drusogenesis is twofold. First, drusen stimulate inflammation. The analysis of drusen revealed the presence of various proteins, including major histocompatibility complex (MHC) class II antigens (Johnson et al. 2000), proteins associated with the activation of the immune system, including β-amyloid, C-reactive protein (CRP) or membrane attack complex—MAC (Anderson et al. 2002, 2004; Mullins et al. 2000). Drusen activated macrophages to clear some of drusen components and to express scavenger receptors (Kamei et al. 2007; Luhmann et al. 2009). The impairment of macrophage-mediated clearance system may result in the overwhelming amount of pro-inflammatory deposits leading to the recruitment of tissue-destructive macrophages and the activation of the complement system. Second, the presence of drusen between two functionally and structurally interacting tissues may hinder the process of oxygen and nutrients delivery to RPE cells by Bruch’s membrane (Strauss 2005). The depletion of these compounds may be an onset ultimately leading to imbalance between pro-angiogenic and anti-angiogenic factors resulting in the neovascularisation and progressing from dry to wet AMD (Frank 1997). It is suggested that the excess of pro-angiogenic compounds such as FGF, TNF-α and matrix metalloproteinases (MMPs) is released by neutrophils, mast cells and macrophages at the site of pro-inflammatory drusen deposites (Kijlstra et al. 2005).

## Oxidative stress in the retina

The generation and neutralization of radicals, molecules with unpaired electron(s) is a physiological process. Provided that radicals are effectively scavenged by the cellular antioxidant defence systems, their presence is not detrimental. The imbalance between oxidants and antioxidants in favour of the oxidants, results in oxidative stress. This is a pathogenic condition leading to damage of numerous cellular components including lipids, proteins and nucleic acids. A concept formulated by Denhama Harman states that aging is a result of ROS-induced damage accumulation (Harman 1956). Experimental data support this thesis as oxidative stress may accelerate the process of aging and may play a role in the pathogenesis of many aging-related diseases including AMD (Chiba et al. 2009).

**Fig. 1:**
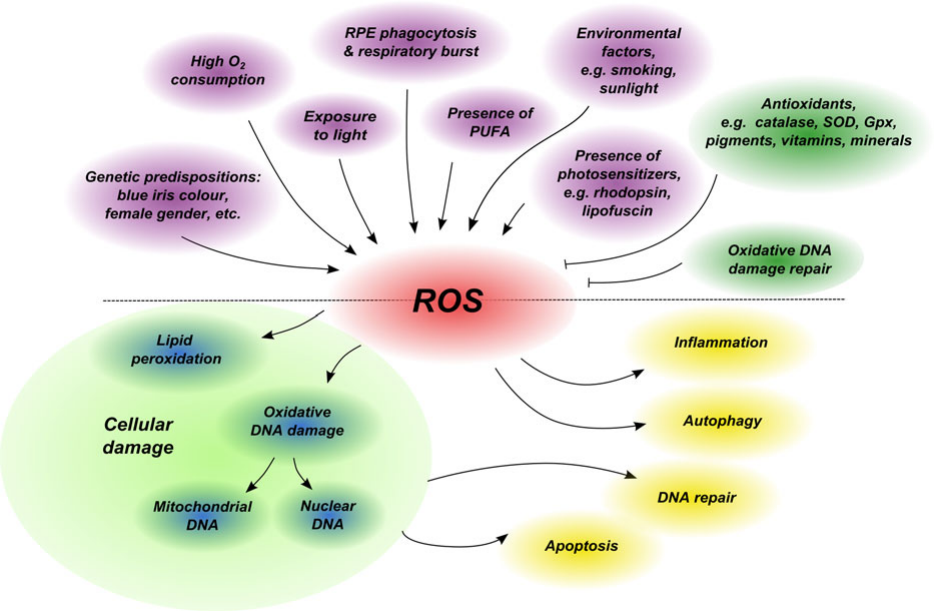
Schematic presentation of ROS involvement in AMD pathology

The retina is a tissue abundant in ROS (Fig. 1). First, the oxygen consumption in the retina is the highest among all human tissues (Yu and Cringle 2001). Second, RPE and photoreceptors are exposed to high-energy light, which is focused in the macula (Youssef et al. 2011). Third, the cell membrane of photoreceptors is rich in polyunsaturated fatty acids (PUFA), which are readily oxidized (Anderson et al. 1974). Fourth, there are many photosensitizers in RPE and photoreceptors (Hunter et al. 2012). Finally, the phagocytosis of POS conducted by RPE cells may be accompanied by a respiratory burst—a rapid eruption of ROS (Miceli et al. 1994). POS which are wearing out contain lipids, proteins and others oxidized particles, the driving force of a respiratory burst, born as a result of exposure to light and oxygen-rich environment in the photoreceptors (Tate et al. 1995).

### Oxygen consumption

Photoreceptors, cells of high metabolic activity, are in a high demand for oxygen and nutrients, which are delivered through blood vessels. Due to the high consumption of oxygen, its supply in the retina is higher than in other tissues (Yu and Cringle 2001). The high partial pressure of oxygen promotes generation of ROS in the retina (Fig. 1). Mitochondria are a major source of ROS and thus they are perturbed by oxidative stress (Cui et al. 2012). This is of a special importance in the context of mitochondrial DNA (mtDNA), which may be more susceptible to oxidative damage than nuclear DNA (Ballinger et al. 1999; Jin et al. 2001). This fact is mainly attributed to the mtDNA proximity to the source of ROS production, the lack of mtDNA protection by histones and other DNA-associated proteins, the lack of introns in mtDNA and the less effective mtDNA repair systems in comparison to nuclear DNA (Desler et al. 2011). That is why mtDNA rapidly accumulates mutations, which further can cause disorder of the respiratory chain function leading to generation of ROS (Cui et al. 2012). Non-dividing cells, including RPE cells, are particularly prone to accumulate mtDNA damage due to their inability to reduce defective mitochondria during mitosis. Changes in mitochondrial number, size, shape, matrix density, cristae architecture and membrane integrity were distinct in RPE cells obtained from donors aged 60 and more when compared to those obtained from younger individuals (<60 years) (He et al. 2010). These mitochondria dysfunctions were associated with low ATP level, attenuated mitochondrial membrane potential, reduced cytoplasmic Ca^2+^ and augmented mitochondrial Ca^2+^ sequestration. Knockdown of MnSOD (superoxide dismutase, which binds manganese), an antioxidant mitochondrial enzyme, stimulated a long-term mitochondrial oxidative stress, which evoked the increase in superoxide anion, apoptotic cell death, degeneration of RPE cells, thickening of Bruch’s membrane, shortening and disorganisation of the photoreceptor outer and inner segments (Justilien et al. 2007).

### Irradiation

Radiation reaching the eye is partly absorbed by the cornea and lens, whereas the rest of it (400–760 nm) penetrates the eye reaching the retina, where it may induce damage to retinal cells (Chalam et al. 2011; Fig. 1). It was demonstrated that the exposure of the retina to blue light (441 nm) in vivo resulted in the damage to POS, the cellular proliferation, the mitotic alterations in the RPE and choroidal cells and the RPE pigment mottling—signs resembling atrophic changes in AMD (Ham et al. 1978). Other study also showed that constant illumination of the retina led to the damage of photoreceptors in vivo (Wiegand et al. 1983). The link between irradiation and oxidative stress was observed when light-induced retinal damage stimulated the expression of oxidative stress-inducible heme oxygenase-1 (HO-1) (Organisciak et al. 1998). The administration of antioxidants before radiation exposure protected the retina from the damage (Organisciak et al. 1998; Ranchon et al. 1999; Lam et al. 1990). Blue light seems to be the most dangerous to RPE, not only because it is the most energetic radiation reaching the monolayer of RPE cells, but also because it promoted photooxidation of lipofuscin generating the reactive photoproducts including-*N*-retinylidene-*N*-retinylethanolamine (A2E), cell apoptosis and DNA oxidation (Sparrow et al. 2000, 2002, 2003; Sparrow and Cai 2001).

### Polyunsaturated fatty acids

Photoreceptor’s membrane is characterised by a unique composition of lipids, predominantly containing PUFAs, with the most abundant representative being docosahexanoic acid (DHA) (22:6 ω3), exclusively of dietary origin. Since the susceptibility of unsaturated fatty acids to oxidation increases with the number of double bonds, the photoreceptors are particularly vulnerable to lipid peroxidation (Witting 1965). This process may produce peroxides and organic radicals, which may cause functional and structural damage to cell membrane resulting in the degeneration of photoreceptors (Anderson and Krinsky 1973; Arstila et al. 1972). Retinal damage was significantly reduced in rats fed a diet deficient in DHA or linoleic acid, a DHA precursor (Bush et al. 1991; Organisciak et al. 1996). The age-dependent susceptibility of the posterior pole retina to lipid peroxidation was observed suggesting the attenuation of antioxidant defence systems with aging (De La Paz and Anderson 1992).

### Photosensitizes

Photosensitizes are chemical compounds that absorb light and subsequently emit radiation, which may induce chemical reactions contributing to cell photochemical damage (Fig. 1). There are a few photosensitizes, including rhodopsin, lipofuscin, melanin and the mitochondrial respiratory enzymes, e.g. cytochrome c oxidase, which were demonstrated to be essential factors for photodamage generation in the retina (Hunter et al. 2012).

The degree of retinal degeneration positively correlated with the rhodopsin content in the retina before light exposure (Rapp and Williams 1979, 1980; Organisciak and Winkler 1994; Organisciak et al. 1991). The susceptibility to light-induced damage ameliorated with age as assessed by the level of recovered rhodopsin after light exposure (Organisciak et al. 1998). Administration of antioxidants, including ascorbate, ascorbic acid and dimethylthiourea (DMTU) led to an inhibition of rhodopsin loss in the retina suggesting oxidative nature of rhodopsin-mediated photodamage (Organisciak et al. 1985, 1990, 1992, 1998). In support of this statement, the DMTU treatment of the light-exposed rats suppressed the induction of HO-1 mRNA encoding oxidative stress-induced enzyme (Organisciak et al. 1998).

Lipofuscin, an aggregate primarily consisting of lipids, proteins and pigment derivatives such as A2E, is progressively accumulated in dysfunctional RPE cells (Delori et al. 2001). It was shown that the illumination of RPE cells with blue light induced the oxygen uptake in an age-dependent manner (Rozanowska et al. 1995). The observed photoreactivity of RPE cells was mainly attributed to lipofuscin, which generated ROS including singlet oxygen, superoxide anion and hydrogen peroxide (H_2_O_2_) under aerobic conditions. This photoinducible generation of radicals was shown to result in lipid peroxidation, partial or complete inactivation of antioxidant enzymes, including SOD and catalase, in RPE cells and RPE cellular dysfunction (Wassell et al. 1999; Shamsi and Boulton 2001). Also, the exposure of A2E to blue-light initiated the production of ROS and induced apoptosis in ARPE-19 cells (Sparrow et al. 2000). However, the illumination of ARPE-19 cells with blue light in the absence of A2E, did not promote cell death. The accumulation of A2E led to the dysfunction of lysosomes in a dose-dependent manner, which is a prerequisite for the pathogenesis of diseases associated with excessive lipofuscin accumulation, including AMD (Holz et al. 1999). The illumination of cells inhibited the cytochrome oxidase activity in the light-intensity-dependent manner in the presence of AE2 in the cells inducing impaired electron flow in the respiratory chain (Shaban et al. 2001).

### Inflammation

Although AMD is not considered a typical inflammatory disease, the pathogenic role of immunologic processes in the occurrence and progression of AMD is well documented. The correlation between immunological/inflammatory gene polymorphisms and AMD indicates the involvement of inflammation and immune-mediated processes—complement activation, in the pathogenesis of this disease (Bergeron-Sawitzke et al. 2009; Ryu et al. 2010). Furthermore, immunocompetent cells, such as macrophages and lymphocytes, were present in the chorioretinal tissues affected by AMD (Penfold et al. 1985; Lopez et al. 1991). Also, the complement pathway was deregulated in eyes from AMD patients. It was demonstrated that oxidative damage induced inflammation and initiated formation of AMD-like lesions, including drusen accumulation next to the RPE layer upon aging, development of lesions mimicking geographic atrophy (GA) in RPE and the blindness in mice, indicating a direct link between oxidative damage and inflammatory response in AMD (Hollyfield et al. 2008). Thus, it may be expected that modulation of the level of oxidative damage may influence the inflammatory response. Also, the relationship showing the stimulation of antioxidant enzyme activity upon administration of acute inflammatory response inductor, endotoxin, in the rabbit eye was manifested (Recasens and Green 1992). Endotoxin administration was associated with a significant SOD induction in choroids and retinas of adult animals, but not of aged animals. This effect may be indicative of the vulnerability of ocular tissues from aged animals to inflammation-related oxidative stress due to their inability to induce SOD in response to an inflammatory stimulus.

## Antioxidant defence

Because of many studies demonstrating a causative role of oxidative stress in the etiopathogenesis of AMD, the antioxidant status in individuals with and without this disease was extensively investigated. Although the majority of studies conducted in vitro and in vivo indicate a protective role of antioxidants, the population-based studies on dietary antioxidant intake lack consistency and reliability. The confounding data are derived from multiple interactions between compounds taken with food. Data collected by the National Health and Nutrition Examination Survey (NHANES) showed that the consumption of fruit and vegetables, being a source of antioxidants, negatively correlated with the occurrence of AMD (Goldberg et al. 1988). However, other factors, including increased concern in healthy lifestyle of vegetarians, were not taken into account in this study.

### Pigments

To protect the eye from the high energy radiation the RPE cells are equipped with the specialised set of various pigments which absorb part of light and thus constitute a radiation filter for cells (Beatty et al. 1999, 2000, 2001). The majority of light is absorbed via melanin present in melanosomes (Boulton 1998). The remaining part of the light spectra is absorbed by photoreceptors and their pigments—carotenoids, lutein and zeaxanthin, which are selectively accumulated in the retina (Bone et al. 1985). These carotenoids are referred to as macular pigment, which is thought to shield photoreceptors from blue light since it reduces the amount of light, which reaches photoreceptors by approximately 40 % (Bone et al. 1997; Snodderly et al. 1984; Fig. 1). The maximum absorption wavelength for A2E is near 450 nm (blue light). High absorption of this range of wavelength (absorbance spectrum peaks at 460 nm) by macular pigment prevents A2E oxidation and subsequent generation of ROS (Haegerstrom-Portnoy 1988; Junghans et al. 2001; Landrum and Bone 2001; Pease et al. 1987). Apart from being a blue light filter, carotenoids manifest their antioxidant properties through quenching reactive oxygen intermediates (Foote and Denny 1968; Krinsky and Deneke 1982). The distribution of these carotenoids was unequal in the retina. Zeaxanthin is abundant in the macula and lutein in the peripheral retina, which suggests different functions of these pigments (Bone et al. 1988, 1997). Carotenoids ability to scavenge free radicals was increased with decreasing oxygen partial pressure as assessed through the measurement of lipid peroxidation in a chemical model, indicating that the efficiency of these ROS scavengers may be modulated by oxygen concentration (Jorgensen and Skibsted 1993). Although it was not demonstrated, it may be supposed that the antioxidant activity of carotenoids may be reduced under oxidative stress. Carotenoids may interact with other antioxidants complementing their function, such as the α-tocopherol regeneration or the enhancement of antioxidant action of vitamin C (Edge et al. 1997; Packer 1993). The majority of studies including population-based case-control and cohort studies indicated a protective effect of lutein and zeaxantine (assessed by plasma or serum level measurements or interview-based intake estimations) in relation to AMD, especially its wet form (Eye Disease Case–Control Study Group 1993; Snellen et al. 2002; Delcourt et al. 2006). However, oppose effects in serum have also been demonstrated (Mares-Perlman et al. 1995). Recent study conducted within the age-related eye disease study 2 (AREDS2) project manifested a high macular pigment optical density (MPOD), indicator of macula health, in individuals supplemented with 10 mg of lutein and 2 mg of zeaxanthin per day, when compared with control group not receiving this supplementation (Bernstein et al. 2012). Concordant results were obtained from the mouse model, DKO mice, which develop focal retinal lesions that had clinical, biochemical, and pathological features of AMD, including the degeneration and atrophy of focal photoreceptors and RPE (lipofuscin accumulation, hypertrophy, and hypotrophy) as well as the presence of some drusenoid deposits (Ramkumar et al. 2010, 2013; Tuo et al. 2007; Zhang et al. 2013). Although this model lacks a macula (like all nonprimate models), it adopts the accumulation of A2E and the degeneration of focal photoreceptor and RPE, which are found in AMD. The supplementation of mice with lutein, zeaxanthin, and long-chain n3 PUFAs: docosahexaenoic acid (DHA) and eicosapentaenoicacid (EPA) demonstrated a benefit of the AREDS2 diet on retinal AMD-like lesions on the clinical and histopathological level including the accumulation of RPE lipofuscin and A2E, the pathologic gene expression as well as the preservation of photoreceptors in comparison to DKO and wild-type mice fed with either normal or with isocaloric diet. The AREDS2 studies conducted on the 1,608 participants showed no reduction in progression to advanced AMD in patients supplemented with lutein and zeaxanthin or DHA and EPA or 4 these compounds in comparison to individuals receiving placebo. It is worth noting that although there is a large body of evidence in favour of the role of lutein in the retina functioning, its participation in the development of AMD remains a matter of debate. The new light was recently shed on the influence of lutein on the inflammation. Data collected from epidemiological studies revealed an inverse dependency between lutein concentration in serum and circulating inflammation markers, such as intercellular adhesion molecule 1 (ICAM-1) and CRP (van Herpen-Broekmans et al. 2004; Seddon et al. 2006). Other study, in which CRP level was also compared, did not confirm this finding (Kritchevsky et al. 2000). This may be attributed to the fact that only healthy individuals, who might have had too low initial level of inflammatory biomarkers, were taken into the examination (Kijlstra et al. 2012). In vivo experiments, conducted on laser-stimulated mouse model of CNV, revealed that the administration of lutein before exposure to laser resulted in the decreased volume of CNV, the inhibited infiltration of macrophages into the CNV area of the retina and the down-regulated expression of pro-inflammatory proteins, including CCL2 (monocyte chemotactic protein-1), vascular endothelial growth factor (VEGF) and ICAM-1 as compared to controls (Izumi-Nagai et al. 2007). Pre-treatment with lutein inhibited the inflammation and therefore the activation of NF-κB, which is one of the factors causing the development of CNV. Moreover, the experiments in vitro demonstrated that lutein exposure blocked TNF alpha- or lipopolysaccharide-induced expression of NF-κB in ARPE-19 cells and macrophages, respectively (Izumi-Nagai et al. 2007; Kim et al. 2008). Thus recent literature indicates three functions of lutein in the retina: a blue light filter, a ROS scavenger and a blocker of inflammatory response.

### Vitamins

Vitamins C and E play an important role as antioxidants in the prevention of AMD. Vitamin C acts as a ROS scavenger and reconstitutes vitamin E, which is anchored in the cell membrane and prevents lipid peroxidation (Beatty et al. 2000; Sies et al. 1992; Fig. 1). The studies on animals showed that the pre-exposure supplementation with vitamin C reduced the rod cell loss and preserved DHA in outer segment of these cells (Organisciak et al. 1985). The deficiency of vitamin E led to the retina degradation, the augmented lipofuscinogenesis in RPE, the decrease in the PUFA level in rod outer segments and RPE as well as the augmented sensitivity of the retina to photo-oxidative damage (Hayes 1978; Farnsworth et al. 1979; Beatty et al. 2000). However, the population-based studies are incoherent in results on the protective action of vitamin C or vitamin E. The supplementation of vitamin C at 500 mg each day or of vitamin E at 400 IU the alternate days (doses applied in the AREDS formulation) had no effect on the incidence of the diagnosis of AMD in 14 236 men aged ≥50 after 8 years of treatment (Christen et al. 2012). This lack of association between vitamin C or vitamin E intake and AMD may be attributed to a weak protective effect of dietary vitamins, which may be below the detection limit or the period of high dose intake of vitamins was too short. Recent report demonstrated that the oral preparation containing lutein, zeaxanthin, vitamin C, vitamin E, copper, and zinc led to the functional and morphologic benefits in patients with early AMD (Beatty et al. 2013). Since an individual diet includes a battery of distinct antioxidants, the assessment of the selected compound is a difficult task. Although it seems that the individual components of the AREDS formulation may evoke a weak protective effect, their combination may be strong enough to provide beneficial effect to the treatment/prophylaxis of AMD.

### Minerals

Minerals may function as regulators of antioxidant enzymes, thus their deficiency may negatively affect the cellular antioxidant defence system (Fig. 1). The alterations in the level of elements in aqueous humor of patients with dry AMD confirmed that the disturbance in mineral homeostasis is associated with AMD (Jünemann et al. 2013). AMD patients had significantly elevated concentration of zinc, cadmium, cobalt and iron. On the other hand, the concentration of copper was diminished in patients with AMD. However, no significant difference was observed for manganese and selenium. Zinc regulates the activity of CuZnSOD and catalase, induces the expression of metallothionein—a cystein-rich protein that provides protection from oxidative stress and interacts with retinol dehydrogenase, which participates in the restoration of retinol in the visual cycle (Tate et al. 1997; Sato and Bremner 1993). The level of zinc was reduced in RPE, choroid complex and neural retina of patients with AMD when compared to control (Erie et al. 2009). The accumulation of lipofuscin was observed in the RPE of rats on a zinc scarce diet (Julien et al. 2011). This was accompanied by the appearance of macrophages in the choroids as well as at Bruch’s membrane, indicating that zinc may participate in the inflammatory response in the retina. The zinc supplementation at 80 mg/day for 2 years protected AMD patients from loss of visual acuity (Newsome et al. 1988). Among zinc-supplemented individuals loss of visual acuity occurred in 3.8 % patients, whereas in patients taking placebo it was 10 %. Zinc is also included in the ARDES formulation—the antioxidant cocktail utilized for AMD treatment. Beside zinc in the cocktail formulation, there is copper, which is included in order to prevent copper deficiency anemia, a condition associated with high level of zinc intake. Similarly as zinc, manganese is a cofactor of antioxidant enzyme, MnSOD. Cadmium, rated as the 8th most toxic substance by the agency for toxicity and disease registry priority list of hazardous substances, accumulates in aging human retinal tissues and its level is doubled in the neural retina and RPE from AMD-affected eyes when compared with controls (Wills et al. 2009; ATSDR 2005). Interestingly, cadmium content was higher in females than males for both control and AMD-affected eyes reflecting the gender differences in the AMD prevalence. It was demonstrated that cadmium might interfere with the metabolism of zinc by binding to the zinc transport proteins (Girijashanker et al. 2008). Since these proteins have high affinity for cadmium thus cadmium may deplete zinc level in the retina through cadmium competition with zinc for these transporters. Pre-treatment of ARPE-19 cells with either manganese or zinc prevented cadmium accumulation in these cells (Satarug et al. 2008). Manganese demonstrated a stronger effect in blocking cadmium uptake than zinc and it induced the expression of HO-1 on mRNA and protein levels suggesting that manganese may enhance resistance to oxidative stress in RPE cells. Apart from zinc, cadmium competes with other metals, including manganese, calcium and iron for metal transporter protein(s) in order to enter the cells (Bannon et al. 2003; Martin et al. 2006; He et al. 2006). We have demonstrated the association between polymorphism in genes encoding enzymes regulating iron homeostasis including transferrin gene and the iron-regulatory protein-1 and -2 genes as well as in the generation and removal of iron-mediated oxidation: *NQO1, NOS3* and *NFE2L2* and the occurrence of AMD (Wysokinski et al. 2013; Synowiec et al. 2012, 2013).We have also found that the serum level of transferrin was higher in AMD patients when compared with those without AMD (Wysokinski et al. 2013). In support of this observation, the level of transferring was increasing during the course of rapidly progressing retinal degeneration in rd10 mice when compared with controls at the same age (Deleon et al. 2009). Furthermore, age-related iron accumulation impaired the phagocytosis and lysosomal functions of RPE cells in the aged rodents—dysfunctions associated with AMD (Chen et al. 2009a). Recent findings showed that iron chelator was protective against the light-induced retinal degeneration and reduced oxidative stress in mouse retina indicating a crucial participation of iron in the generation of oxidative stress in the retina (Song et al. 2012). Selenium is an activator of glutathione peroxidase (Gpx) (Singh et al. 1984). Currently undergoing clinical trial SELECT examining the protective effect of selenium in AMD in men should clarify whether this element plays a role in the pathogenesis of AMD. Regardless of this trial, selenium inhibited VEGF production in the epithelial cancer cells in vitro (Jiang et al. 2000). Thus it is possible that selenium could also participate in the regulation of angiogenesis in the eye impeding the development of wet AMD.

### Enzymatic antioxidants

Apart from components, which are provided with diet, inherent antioxidant compounds including antioxidant enzymes play a crucial role in maintaining oxidative balance. Enzymatic antioxidants are the most potent scavengers of ROS when compared with small molecular weight antioxidants. The importance of antioxidant enzymes in maintaining cell physiology was demonstrated when the intentionally introduced imbalance in their level stimulated different phenotypes. The increase in MnSOD or FeSOD sensitized *E. coli* cells to paraquat, whereas the increase in CuZnSOD rendered HeLa cells resistant to this compound (Scott et al. 1987; Bloch and Ausubel 1986; Elroy-Stein et al. 1986). In accordance with these findings, the increase in CuZnSOD sensitized mouse epidermal cells JB6 to the formation of DNA strand breaks, the growth inhibition and the cell death in the presence of O_2_^−^ or H_2_O_2_ (Amstad et al. 1991). The compensatory effect was observed when glutathione peroxidise was added, indicating that the slight deviations in balance between antioxidant enzymes may influence the oxidation-induced genome instability and cell death. At least three enzymes i.e. superoxide dismutase, catalase, and Gpx, that protect the retina from oxidative damage are present in RPE cells and photoreceptors. The supplementation of low molecular antioxidants may be applied in the treatment of AMD, but it seems that it plays a supportive role and rather alleviates ailments than cures the disease. However, the restoration of function or expression of genes encoding antioxidant enzymes may be much more effective. The treatment based on the re-establishment of antioxidant enzymes balance may be a way to treat AMD. Additionally, the examination of individual genetic predisposition may prevent initiation and progression of AMD as well as serve for treatment purposes.

#### Superoxide dismutase

SOD catalyzes the dismutation of superoxide into oxygen and H_2_O_2_ with catalytic efficiencies near the diffusion limit (McCord and Fridovich 1969; Ragsdale 2009). Since the reaction is limited only by the frequency of collision between the enzyme and superoxide, thus SOD serves a key antioxidant role. The importance of SOD is manifested by the severe pathologies associated with lack of this enzyme in mouse models (Lee et al. 2013; Kliment et al. 2009; Behndig 2008). There are two major families of superoxide dismutases, depending on metal cofactor: CuZnSOD (SOD1) in cytoplasm and MnSOD (SOD2) in mitochondria in humans (Yu 1994). The role of SOD in the development of AMD is a matter of debate due to the inconsistency of results. Treatment of ARPE-19 cells with acrolein, a potent source of oxidative stress and mitochondrial dysfunction, resulted in a decreased SOD activity as compared with control (Liu et al. 2007; Jia et al. 2007; Feng et al. 2010). The treatment of ARPE-19 cell with α-tocopherol did not influence SOD activity, however, the pre-treatment with this form of vitamin E followed by the subsequent exposure to acrolein led to the protection of SOD activity (Feng et al. 2010). The up-regulation of SOD1/2 expression resulted in oxidative damage in RPE cells as assessed by the measurement of protein carbonyl group content—a marker of protein oxidative damage (Lu et al. 2009). Although the studies conducted in vitro coherently indicate the role of SOD in oxidative stress responses they do not clearly show its association with AMD. The immunoreactivity of SOD in cytoplasm and lysosomes tended to increase with age in macular RPE cells with and without wet AMD (Frank et al. 1999). However, SOD activity of the RPE periphery tended to decline with age, indicating that the distribution of SOD may change during aging in the retina (De La Paz et al. 1996). An increased level of SOD in serum in AMD patients was demonstrated on two distinct Chinese populations (Shen et al. 2012; Jia et al. 2011). Also, differences were shown in the genotype distribution of the p.Ala9Val polymorphism in the *MnSOD* gene, which was associated with the level of MnSOD mRNA and protein expression in blood, between patients with AMD and controls (Kowalski et al. 2010). On the other hand, a high level of erythrocyte SOD activity was not associated with AMD in a population-based cross-sectional POLA study (Delcourt et al. 1999). However, increase in SOD activity correlated with twofold increase in nuclear cataract. This study is of a special significance since some forms of cataract are associated with the increased risk of AMD (Klein et al. 2012). The most convincing results confirming the role of SOD in the pathogenesis of AMD come from the report in which the level of SOD was examined in RPE cells obtained from human donors (Liles et al. 1991). In this study, SOD activity showed no significant correlations with aging or AMD.

#### Glutathione reductase and glutathione peroxidase

Glutathione (GSH) is an antioxidant and participates in H_2_O_2_ decomposition, which may be catalysed by selenium-stimulated Gpx. Some studies demonstrated an age-related decrease in plasma GSH, whereas the level of glutathione disulfide (GSSG), oxidised state of GSH, in whole blood increased with age (Samiec et al. 1998; Kretzscharm and Muller 1993; Lang et al. 1992). GSH efflux may contribute to oxidative stress due to GSH depletion. Treatment of RPE cells with α-crystallins (αA and αB, normally present in the retina and serving a protective function) rendered them resistant to oxidant-induced cell death (Sreekumar et al. 2012). This correlated with a twofold increase in the concentration of GSH. On the other hand, a decrease in GSH was observed in cells lacking αA and αB, suggesting a causative role of αA and αB in the regulation of GSH level. High level of GSH may be a consequence of at least two processes: an enhanced GSH biosynthesis and a higher conversion of GSSG to GSH by glutathione reductase. An increase in α-crystallin level accompanied an increase in MRP1 expression—a member of multidrug resistance protein family. MRP1 inhibition decreased GSH efflux, accelerated the intracellular level of GSH and GSSG and made RPE cells resistant to oxidative stress-induced cell death. These results show that the resistance to oxidative stress is executed via α-crystallins-mediated regulation of GSH level. Since it is controlled by glutathione reductase and Gpx, changes in the activity of these enzymes may affect the cellular response to oxidative stress and thus participate in the pathogenesis of AMD. Indeed, blood glutathione reductase activity was lowered in patients with AMD compared with controls (Cohen et al. 1994). Interestingly, Gpx increased activity was found in retinal homogenates of cynomolgus monkeys with early-onset AMD (Nicolas et al. 1996). Additionally, the higher level of plasma Gpx was associated with a nine-fold increase in the prevalence of late AMD, but not early AMD in POLA study conducted on 2,584 subjects (Delcourt et al. 1999). In accordance with previous results, the illumination of rats with white fluorescent light for 24 h resulted in an increased level of Gpx in the eye as revealed by immunohistochemistry study (Ohira et al. 2003). Gpx was up-regulated in POS and RPE at the posterior retinal pole whereas the peripheral retina showed a low change in Gpx level. The high expression of Gpx in the RPE was maintained until day 7 after illumination. The Gpx level decreased on day 1 after illumination and was not observed on day 3 or 7 after the light exposure in POS. The increased expression of Gpx1 or Gpx4 reduced the oxidative stress in RPE cells as assessed by the measurement of protein carbonyl group content (Lu et al. 2009). Furthermore, double over-expression of Gpx4 and SOD1 or SOD2 protected RPE cells from oxidative stress generated by the increased level of SOD1 or SOD2. The presence of Gpx4 or Gpx1 reduced the amount of protein carbonyl group in RPE cells treated with oxidative stress-generating factors, including paraquat, H_2_O_2_ or hyperoxia. The cells with up-regulated Gpx4 or Gpx1 demonstrated an increased viability against paraquat or H_2_O_2_. The up-regulation of Gpx4 protected retinal structure and function in paraquat, H_2_O_2_ or hyperoxia models of oxidative-damage-induced retinal degeneration in transgenic mice with inducible expression of Gpx4 in photoreceptors. Gpx4 was required for regular maturation of photoreceptors in mice (Ueta et al. 2012). Mitochondria were the prime source of Gpx4 expression in wild-type retina. Photoreceptors developed and differentiated regularly until postnatal day 12 and then underwent degeneration and disappeared by postnatal day 21 in mice with *Gpx4* knockdown. Therefore, the increase in Gpx activity is associated with AMD. Thus it seems that RPE cells try to dispose of overwhelming amount of H_2_O_2_ which is generated in the course of AMD development.

#### Catalase

Catalase is an iron-dependent enzyme that has two distinct functions—it may act catalytically or peroxidatively (Chance et al. 1979; Halliwell and Gutteridge 1985). Catalase activity has been shown to decrease in both macular and peripheral RPE during aging (Liles et al. 1991). The immunoreactivity of catalase in cytoplasm and lysosomes showed an age-dependent reduction in macular RPE cells of eyes with and without AMD (Frank et al. 1999). No correlations between aging and catalase activity (De La Paz et al. 1996) or the expression of catalase mRNA were observed (Miyamura et al. 2004). Thus the catalase decline associated with AMD may be age-independent and the catalase activity may be irrespective of its mRNA level suggesting that the AMD-related silencing of catalase activity may be conducted during translation at the earliest. The treatment of RPE cell with ROS-generating compounds stimulated expression of catalase (Tate et al. 1995; Miceli et al. 1994). The inhibition of catalase during ROS uptake increased thiobarbituric acid-reactive substances (TBARS), a byproduct of lipid peroxidation, by 66 % in RPE cells (Miceli et al. 1994). Additionally, the transduction of RPE cells in vitro and in vivo with catalase gene resulted in a protection of transduced cells from H_2_O_2_, as well as the adjacent RPE cells or photoreceptors without up-regulated catalase expression (Rex et al. 2004). These results indicate the protective role of catalase against oxidative stress in RPE and neighbouring cells. The presence of ROS-inducing compounds led to a down-regulation of catalase expression at the transcript and protein levels which was accompanied by an enhanced expression of miR-30b, a member of the miR-30 family (Haque et al. 2012). In addition, ROS-generating agents induced transient methylation of the CpG island II in the calatase promoter in hepatocellular carcinoma (HCC) cell line (Min et al. 2010). Furthermore, the treatment of cells with the antioxidant or with the DNA methyltransferase 1 (DNMT1) inhibitor demethylated the catalase promoter and restored the expression of catalase. The catalase expression and activity was modulated by the level of transcription factor Oct-1, which expression was inhibited through DNA methylation in the presence of ROS (Quan et al. 2011; Tantin et al. 2005). Although these studies were conducted on HCC cells, the mechanism of catalase regulation may be similar in RPE cells. Also the catalase expression was modulated by the acetylation of histone H4 in leukemia cells, indicating that the regulation of catalase expression is a complex and elusive mechanism involving at least three pathways (Lee et al. 2012).

## Regulation of antioxidant enzyme expression via microRNA and transcription factors

The study on the role of miRNA in AMD was prompted by the observations that (1) the miRNA expression pattern differs between AMD patients and individuals without this disease (Kutty et al. 2010) and (2) the administration of synthetic RNA may regulate endogenous miRNA expression and hereby restore the expression pattern of physiological state, arresting or even retreating the development of the disease. Due to these reasons the concern of miRNA in AMD is still growing and may be a potent tool in AMD treatment. miR-30b was over-expressed in ARPE-19 cell under a sublethal dose of oxidative stress (Haque et al. 2012). The administration of the miR-30b antagomir reversed the effect stimulated by the miR-30b mimic—increased the expression of catalase even under oxidant environment. miR-23a, a member of the miR-23~27~24 cluster, was down-regulated in human RPE cells of AMD patients (Lin et al. 2011). After the H_2_O_2_ treatment the level of miR-23a was reduced in RPE cells. The over-expression of miR-23a protected RPE cells from H_2_O_2_-induced apoptosis, but had no effect on the cell viability under normal conditions. miR-23a directly targeted Fas—a protein participating in ROS-evoked apoptosis—as shown by the attenuation of H_2_O_2_-induced up-regulation of Fas accompanying the over-expression of miR-23a. The expression of Fas and FasL was increased in the photoreceptor and RPE layer of the choroidal neovascular membranes from AMD patients (Hinton et al. 1998; Dunaief et al. 2002).

In particular, miRNA processing in the cell may be impaired in AMD interfering with the expression of antioxidant enzymes. In GA—the advanced form of AMD resulting from the death of RPE cells—the miRNA-processing enzyme Dicer1 was depleted in RPE cells (Kaneko et al. 2011). The down-regulation of Dicer1 resulted in the degeneration of RPE cells in mice. This effect was not observed for other miRNA-processing enzymes, including Drosha, DGCR8 or Ago2. The knockdown of *Dicer1* induced the accumulation of cytotoxic *Alu* RNA. Interestingly, the level of *Alu* RNA was increased in GA patients. The treatment with antisense inhibitor of *Alu* RNA prevented Dicer1 down-regulation, which correlated with Dicer1-mediated degradation of *Alu* RNA in humans and prevented RPE degradation in mice. Recent findings, carried out on animal models, indicated a role of endoplasmic reticulum (ER) stress in the retinal degeneration accompanied by oxidative stress and death of photoreceptors (Lin et al. 2007; Yang et al. 2007, 2008). XBP1 is a transcription factor regulating ER stress. Its down-regulation enhanced the photoreceptor degradation in *Drosophila* model for autosomal dominant retinitis pigmentosa (Ryoo et al. 2007). The activity of XBP1 was reduced in RPE cells with a light-induced retinal degeneration in vivo (Zhong et al. 2012). The siRNA-mediated knockdown of *XBP1* correlated with the decreased expression of SOD1, SOD2, catalase and glutathione synthase along with the increased susceptibility of RPE cells to oxidative damage (Zhong et al. 2012). The expression of SOD1, SOD2 and catalase was reduced in the RPE cells deprived of XBP1 and accompanied by the increase in oxidative stress in the mouse line in comparison with wild type counterparts. The RPE cell death reduced the number of cone photoreceptors and the thickening of outer nuclear layer and Bruch’s membrane, shortened POS as well as decreased retinal function in the XPB1-lacking mice.

The response of cells to oxidative stress may include the activation of genes with the antioxidant responsive element (ARE) via the transcription factor Nrf2 (NF-E2-related factor 2) (Lee et al. 2003a). ARE is a *cis*-acting regulatory element of genes encoding phase II detoxification enzymes and antioxidant proteins, such as NAD(P)H quinine oxidoreductase 1 (NQO1), glutathione S-transferases (GST) and glutamate-cysteine ligase (GCL) (Lee et al. 2003a). The function of Nrf2 and the subsequent activation of its target genes were shown to play an important role in cell protection against oxidative stress. Nrf2 is a ubiquitous protein, expressed in numerous cell types regulating a battery of ARE-dependent genes (Lee et al. 2003a, b; Shih et al. 2003; Chen et al. 2009b). The activation of Nrf2 is conducted via electrophiles and oxidants which modify critical cysteine thiols of Keap1 (Kelch-like erythroid cell-derived protein with CNC homology-associated protein 1) thus preventing the Nrf2 ubiquitination and degradation. The *Nrf2* over-expression was protective against toxic phenotype caused by the dominant mutation in *SOD1* in astrocytes (Vargas et al. 2008). Therefore, it may be speculated that increasing the Nrf2 concentration may be beneficial in diseases associated with SOD dysfunction, including AMD. This suggests a dominant role of the regulation of oxidative stress induced genes either via miRNA or transcription factors, which may further serve for AMD treatment purposes. Also other mechanisms cannot be excluded in this regard. The advantage of gene expression based therapy is its extensive efficiency resulting from the opportunity of targeting multiple genes of one metabolic pathway.

Autophagy is a conserved lysosomal pathway responsible for the turnover of malfunctioning proteins in eukaryotic cells. The accumulation of long-lived proteins, excess or damaged organelles, and aggregation-prone proteins may be particularly detrimental in the post-mitotic cells such as RPE. The pathogenesis of AMD involves the impairment of protein degradation in RPE cells. The p62/SQSTM1 protein recognizes toxic cellular waste, which is directed to autophagy. The loss of autophagy caused p62 accumulation and the induction of antioxidant proteins including NQO1 and GSTs in mice (Komatsu et al. 2007, 2010). Thus it seems that p62 may be a key inducer of Nrf2 target genes. The p62 bound to Keap1 in a pocket overlapping with the binding pocket for Nrf2. The excess of p62 prevented Nrf2 binding to Keap1 and thus also Nrf2 ubiquitination resulting in the activation of Nrf2 and induction of ARE-dependent genes (Jain et al. 2010; Komatsu et al. 2010; Lau et al. 2010). The deregulation of Nrf2-Keap1 binding may play a role in autophagy-related pathologic conditions as it was shown in mice in which loss of Nrf2 decreased but loss of Keap1 intensified the liver injury observed in autophagy-deficient mice. The p62 is over-expressed in many neurodegenerative diseases such as in Parkinson, Alzheimer, and Huntington’s diseases (Kuusisto et al. 2001, 2002; Nagaoka et al. 2004; Zatloukal et al. 2002). Interestingly, several proteins identified in the deposits occurring in Alzheimer disease have also been found in eye samples isolated from patients with AMD (Mullins et al. 2000). In addition, knockdown of *p62* suppressed autophagy in ARPE-19 cells revealing its important role in proteolysis (Viiri et al. 2010).

Although AMD is not considered a classic inflammatory disease, the pathogenic role of immunologic processes in the occurrence and progression of AMD is experimentally and clinically documented. Correlations between immunological/inflammatory gene polymorphisms and AMD indicate the involvement of inflammation and immune-mediated processes (complement activation) in the pathogenesis of this disease (Bergeron-Sawitzke et al. 2009; Ryu et al. 2010). Furthermore, immunocompetent cells, such as macrophages and lymphocytes, were present in the chorioretinal tissues affected by AMD (Penfold et al. 1985; Lopez et al. 1991). Also, the complement pathway was deregulated in eyes from AMD patients. It was demonstrated that oxidative damage induced inflammation and initiated formation of AMD-like lesions, including drusen accumulation below the RPE upon aging, development of lesions mimicking geographic atrophy in RPE and the blindness in mice, indicating a direct link between oxidative damage and inflammatory response in AMD (Hollyfield et al. 2008). Thus, it may be expected that modulation of the level of oxidative damage may influence the inflammatory response. Also, the reverse relationship showing the stimulation of antioxidant enzyme activity upon administration of acute inflammatory response inductor, endotoxin, in the rabbit eye was manifested (Recasens and Green 1992). Endotoxin administration was associated with a significant SOD induction in choroids and retinas of adult animals but not of aged animals. This effect suggests vulnerability of ocular tissues from aged animals to inflammation-related oxidative stress due to their inability to induce SOD in response to inflammatory stimulus.

## Genetic variability in antioxidant enzymes in AMD

There is a growing body of evidence that oxidative stress may contribute to the initiation and progression of AMD. It is believed that the degeneration of RPE cells is an early event in the pathogenic process leading to AMD. Certain factors affecting RPE cells may render them especially susceptible to oxidative damage. These factors include deficiency in low molecular weight antioxidants, DNA repair and antioxidant enzymes (Fig. 1). In the case of antioxidant enzymes and DNA repair enzymes, their dysfunction cannot be easily reversed. It seems that antioxidant enzymes play a first line of defence against oxidative injury, thus the re-establishment of their balance in cell may be a priority. The application of compounds functioning as enhancers of the antioxidant enzymes expression or activity may constitute a class of potential drugs. The protective effect of compounds on ARPE-19 against t-BH-stimulated oxidative stress was reported for phenol derivative—canonol (Dong et al. 2011). Canonol showed virtually no cytotoxicity, inhibited the t-BH-induced intracellular ROS generation and the ARPE-19 cells death and enhanced the expression of a number of genes encoding antioxidant enzymes, including catalase and glutathione S-transferase-pi (GST-pi). Genetic variability and mutations occurring in DNA sequence encoding antioxidant enzymes may influence the activity and structure of these enzymes, and even the sensitivity of patients to drugs or supplements. The study on the genetic polymorphism Pro198Leu and the variability on alanine repeat codons in the *Gpx*-*1* gene in human lymphoblast cell lines showed a significant variability in the sensitivity of the cells to selenium supplementation (Zhuo et al. 2009). In response to selenium, all human lymphoblast cell lines showed increase in the activity of Gpx ranging from 1.3 to 6.9-fold. Allelic variation in alanine repeat codons of *Gpx*-*1* had an influence on the Gpx-1 thermostability and selenium supplementation changed this parameter depending on the haplotype of the cell line. A C to T substitution at the -9 position in the mitochondrial targeting sequence of the *MnSOD* gene (V16A, rs4880) was associated with the exudative form of AMD in Japanese population (Kimura et al. 2000). This polymorphism resulted in a more efficient transport of MnSOD into the mitochondria and in a higher basal activity of MnSOD, which led to the excessive hydrogen peroxide production. However, this initially reported association was not replicated by other groups (Gotoh et al. 2008; Kondo et al. 2009). However, the p.Ala9Val polymorphism in the *MnSOD* gene was associated with the level of mRNA and protein expression in humans (Kowalski et al. 2010). The Ala9Ala genotype and the alanine allele were more frequent in AMD patients than in healthy subjects. Healthy controls that are homozygotes Val/Val and heterozygotes Ala/Val showed lower expression of MnSOD gene as compared to homozygote Ala/Ala. The lowest expression of MnSOD was noted in homozygotes Val/Val in wet AMD and heterozygotes Ala/Val in its dry form. The multiple analysis of polymorphic sites in the *MnSOD* (rs1799725, rs2758330, rs1967802)*, CAT* (rs480575, rs1408035, rs769217, rs2284367) and *Gpx1* (rs3448, rs3811699) genes conducted on a Northern Irish population showed no significant association with AMD (Esfandiary et al. 2005). Thus, these results manifest the need for further studies concerning polymorphic variability in antioxidant enzymes genes, which may serve as a predictive tool and target for AMD personal therapy.

### The treatment/prophylaxis of AMD with vitamins, minerals and enzymatic antioxidants

Antioxidant supplementation has provided promising results slowing down AMD progression and preventing AMD occurrence indicating its clinical potential. The AREDS study demonstrated that daily oral supplementation with antioxidant vitamins and minerals reduced the risk of developing advanced AMD by 25 % after 5 years (Age-Related Eye Disease Study Research Group 2001). AREDS formulation including zinc (80 mg), vitamin C (500 mg), vitamin E (400 IU), β-carotene (15 mg) and copper (2 mg) has become the major nutritional support for AMD treatment and is routinely recommended to patients being at a high risk of developing advanced AMD in the US (Kowluru and Zhong 2011). Copper is included into AREDS formulation in order to prevent copper deficiency anemia, a condition associated with high levels of zinc intake (Age-Related Eye Disease Study Research Group 2001). Despite that achieving the levels of vitamins and minerals provided in AREDS formulation is difficult with diet alone, diet rich in vegetables lowered the risk of developing AMD (Sommerburg et al. 1998; Seddon et al. 1994). Although no side effects were demonstrated from antioxidant dietary supplementation, they were present in patients taking AREDS formulation indicating that the intake of AREDS formulation has to be preceded with medial consultation and thoughtful consideration. These side effects included (1) minor, clinically non-relevant inconvenience—the skin yellowing due to high intake of β-carotene, (2) serious medical problem associated with the urinary tract due to zinc treatment and (3) severe disorders such as higher risk of occurring β-carotene-associated lung cancer in smokers (Age-Related Eye Disease Study Research Group 2001; Age-Related Eye Disease Study 2 Research Group 2013). Although some data suggest that increased dietary intake of long-chain PUFAs reduces the risk of advanced AMD, however, the addition of lutein/zeaxanthin (carotenoids), omega-3 long-chain PUFAs (DHA and EPA), or both to AREDS formulation did not evoke a significant reduction in progression to advanced AMD (Chong et al. 2008; SanGiovanni et al. 2007; Age-Related Eye Disease Study 2 Research Group 2013). However, lutein/zeaxanthin could constitute an appropriate substitute for β-carotene, which is associated with increased incidence of lung cancer in smokers. Apart from vitamins and minerals, antioxidant enzymes-based therapy seems to be a promise for diseases associated with increased ROS production. Although not yet studied in AMD, promising results obtained from other ROS-associated diseases suggest their potential for AMD treatment. This approach is still in its early stage, struggling with preserving the activity of enzymes and directing enzymes to the target side. This strategy is currently under investigation for treatment of different ROS-associated diseases including atherosclerosis, hypertension, heart failure, diabetes mellitus using extracellular superoxide dismutase (EC-SOD) (Maksimenko and Vavaev 2012). Also, covalent conjugate SOD-CHS-CAT (CHS, chondroitin sulphate) which showed high efficacy and safety is a promising drug candidate (Maksimenko et al. 2004). Although the antioxidant enzymes preserve their activities in vitro there are still problems with their delivery minimizing the risk of proteolysis. New approach exploiting the non-polymeric magnetic nanoparticles as the antioxidant enzymes carrier was successful in endothelial delivery of SOD and catalase preserving their biological activity (Chorny et al. 2010). The antioxidant enzyme-oriented treatment includes also genetic therapy which was proved efficient for genetic transfer of EC-SOD, which ameliorated endothelium function and decreased the arterial pressure in spontaneously hypertensive rats (Chu et al. 2003). The global regulation of the epigenome, which might enhance oxidative stress resistance, is an attractive approach to AMD treatment. Indeed, the synthetic peptide induced the expression of genes repressed due to age-related condensation of euchromatic chromosome regions in people aged 76–80 years (Khavinson et al. 2003). The traditional antioxidant-rich nutrition along with additional antioxidant supplementation supported with new antioxidant enzymes delivery approach seems to be a promising treatment perspective in future.

## Conclusions and future perspectives

AMD is a complex, degenerative and progressive eye disease that usually does not lead to complete blindness but can result in severe loss of central vision. All AMD risk factors such as age, genetics, diet, smoking, oxidative stress and many cardiovascular associated risk factors involve increased ROS production. Therefore, effective ROS scavenging may be essential for preventing AMD. Initial successes with diet supplementation with small molecular weight antioxidants in the AREDS study prompt for considering other elements of antioxidant defence as possible targets in AMD prevention and future therapy. The activity of antioxidant enzymes, the main component of antioxidant defence, depends on the sequence of their genes and their epigenetic profile. Therefore, determination of the genotype of these genes in individuals at AMD risk, may have diagnostic and prognostic significance. Also drugs modifying epigenetic pattern of these genes may be considered in AMD prevention. Such kind of drugs were approved for treatment of some cancers. Manipulating in the sequence of these genes by gene therapy may be considered in extreme cases to prevent progression of AMD, but it seems unlikely that this might reverse pathological changes associated with the disease.
